# Chronic Isolation Stress Affects Central Neuroendocrine Signaling Leading to a Metabolically Active Microenvironment in a Mouse Model of Breast Cancer

**DOI:** 10.3389/fnbeh.2021.660738

**Published:** 2021-07-09

**Authors:** Alessandra Berry, Barbara Collacchi, Sara Capoccia, Maria Teresa D'Urso, Serena Cecchetti, Carla Raggi, Paola Sestili, Eleonora Aricò, Giada Pontecorvi, Rossella Puglisi, Elena Ortona, Francesca Cirulli

**Affiliations:** ^1^Center for Behavioral Sciences and Mental Health, Istituto Superiore di Sanità, Rome, Italy; ^2^Animal Research and Welfare Center, Istituto Superiore di Sanità, Rome, Italy; ^3^Microscopy Area, Core Facilities, Istituto Superiore di Sanità, Rome, Italy; ^4^National Centre for the Control and the Evaluation of Medicines, Istituto Superiore di Sanità, Rome, Italy; ^5^FaBioCell, Core Facilities, Istituto Superiore di Sanità, Rome, Italy; ^6^Center for Gender-Specific Medicine, Istituto Superiore di Sanità, Rome, Italy

**Keywords:** social isolation stress, HPA axis, NPY/AgRP, leptin, brown adipose tissue, depression, mouse model, breast cancer

## Abstract

Social isolation is a powerful stressor capable of affecting brain plasticity and function. In the case of breast cancer, previous data indicate that stressful experiences may contribute to a worse prognosis, activating neuroendocrine and metabolism pathways, although the mechanisms underlying these effects are still poorly understood. In this study, we tested the hypothesis that chronic isolation stress (IS) may boost hypothalamic–pituitary–adrenal (HPA) axis activity, leading to changes in the hypothalamic expression of genes modulating both mood and metabolism in an animal model of breast cancer. This centrally activated signaling cascade would, in turn, affect the mammary gland microenvironment specifically targeting fat metabolism, leading to accelerated tumor onset. MMTVNeuTg female mice (a model of breast cancer developing mammary hyperplasia at 5 months of age) were either group-housed (GH) or subjected to IS from weaning until 5 months of age. At this time, half of these subjects underwent acute restraint stress to assess corticosterone (CORT) levels, while the remaining subjects were characterized for their emotional profile in the forced swimming and saccharin preference tests. At the end of the procedures, all the mice were sacrificed to assess hypothalamic expression levels of Brain-derived neurotrophic factor (*Bdnf*), Neuropeptide Y (*NpY*), Agouti-Related Peptide (*AgRP*), and Serum/Glucocorticoid-Regulated Protein Kinase 1 (*SgK1*). Leptin and adiponectin expression levels, as well as the presence of brown adipose tissue (BAT), were assessed in mammary fat pads. The IS mice showed higher CORT levels following acute stress and decreased expression of *NpY, AgRP*, and *SgK1*, associated with greater behavioral despair in the forced swimming test. Furthermore, they were characterized by increased consumption of saccharin in a preference test, suggesting an enhanced hedonic profile. The IS mice also showed an earlier onset of breast lumps (assessed by palpation) accompanied by elevated levels of adipokines (leptin and adiponectin) and BAT in the mammary fat pads. Overall, these data point to IS as a pervasive stressor that is able to specifically target neuronal circuits, mastered by the hypothalamus, modulating mood, stress reactivity and energy homeostasis. The activation of such IS-driven machinery may hold main implications for the onset and maintenance of pro-tumorigenic environments.

## Introduction

Breast cancer is one of the most common cancers among women and a leading cause of mortality with over 2 million new cases worldwide diagnosed in 2018 (Bray et al., 2018). Although most cancer research is mainly focused on tackling molecular and cellular pathways (microenvironment), there is now clear evidence that interactions of an individual with their physical and social environment (macroenvironment) can influence disease progression (McEwen, 2012; Borgi et al., 2020). Stressful conditions, in particular, are a recognized risk factor for cancer in the clinic and are linked to breast cancer aggressiveness in animal models (Hermes et al., 2009; Volden et al., 2013). Animal studies of breast cancer consistently show that exposure to stress potentiates tumor growth and metastasis (Hermes et al., 2009; Williams et al., 2009; Madden et al., 2013; Volden et al., 2013).

Stress response begins in the brain with the perception and elaboration of external challenges and affects the brain as well as the rest of the body through plastic changes, leading to adaptation. The connection between central stress responses and peripheral target organs triggers a cascade of events involving the activation of a number of neurochemical and inflammatory mediators, such as cytokines, chemokines, and growth factors, modifying cell function/survival, metabolism and behavior, in order to best deal with a stressful challenge (Hayley et al., 2005; Cirulli and Alleva, 2009; Capoccia et al., 2013). Indeed, the stress response involves the activation of the autonomic nervous system and the hypothalamic–pituitary–adrenal (HPA) axis, increasing the secretion of catecholamines and glucocorticoids (GCs), respectively. While the coordinated activation of the stress machinery is pivotal in the short run (acute conditions) to cope with external threats yet, over longer time intervals (chronic stress), it imposes a cost, rendering the organism more vulnerable to pathological conditions (McEwen, 2007). Indeed, chronic stress has been associated to the onset/precipitation of psychiatric disorders and to altered immune responses possibly affecting neoplastic progression both in humans and animal models (Cavigelli et al., 2008). In particular, it is thought to dysregulate immune function through (i) the suppression of protective immunity, (ii) the enhancement of immunosuppressive mechanisms and (iii) the induction/exacerbation of chronic inflammation (Dhabhar, 2009, 2014, 2018; Antoni and Dhabhar, 2019; Zhang et al., 2020).

Cao et al. (2010) in a very intriguing study, provided evidence that environmental/social enrichment is able to reduce tumor growth and increase remission in mouse models of melanoma and colon cancer. Mice exposed to an enriched environment were characterized by increased hypothalamic Brain-Derived Neurotrophic Factor (*Bdnf*) levels associated to decreased leptin and increased adiponectin production in the white adipose tissue (WAT) (Cao et al., 2010; Cao and During, 2012; Liu et al., 2014). Mammary fat is the most abundant component of the breast and is the most probable candidate to affect tumor behavior because adipocytes produce hormones, growth factors, and adipokines. There is increasing evidence that adipokines, such as adiponectin and leptin, secreted by peri-tumoral adipose tissue are involved in several tumors, such as breast cancer (Miyoshi et al., 2006; Schäffler et al., 2007). Adipokines are a heterogeneous class of molecules produced prevalently by WAT that have emerged as modulators of inflammation and immune responses. In addition, they also act as messengers to communicate to the brain the peripheral metabolic status (Schäffler et al., 2007).

Moreover, and most intriguingly, adipokines are emerging as depression biomarkers, and changes in their circulating levels have been related to the severity of depressive symptoms, particularly in young women (Carvalho et al., 2014; Everson-Rose et al., 2018; Syk et al., 2019). Indeed, depressive symptoms often accompany patients with breast cancer as a result of situational fear related to diagnosis and prognosis (Raison and Miller, 2003). In this regard, it is worth noticing that the tumor itself can produce depressive symptoms by triggering systemic inflammatory responses generating a positive feedback loop between coping style and cancer progression (Sephton et al., 2009). Such mechanisms may be greatly affected by environmental conditions, with stressful events overall decreasing the ability to cope with cancer.

While an enriched environment was shown to reduce tumor growth and increase remission (Cao et al., 2010), in animal models of cancer, we have previously shown that social isolation leads to reduced BDNF levels, and increased anxiety and depressive-like behaviors, accompanied by higher levels of corticosterone, all conditions that might favor cancer progression (Berry et al., 2012). BDNF is a neurotrophin highly expressed in the hippocampus and in the hypothalamus, whose levels are decreased as a result of excessive exposure to GCs (Tapia-Arancibia et al., 2004; Price et al., 2018). It plays a key role in neuronal plasticity and the modulation of emotionality as well as in the integration of neuroendocrine and metabolic pathways in response to stressful challenges (Cirulli and Alleva, 2009). Indeed, social isolation, which is commonly experienced by patients with cancer, may represent a critical condition exacerbating depressive symptoms and promoting tumor activity (Pantell et al., 2013; Sumis et al., 2016). People who experience isolation stress (IS) show a worse prognosis and present an elevated chance to die from cancer, such as breast cancer, compared with individuals with a richer social life (Berkman et al., 2004; Kroenke et al., 2006). Worth noticing is that while research on the effects of different stressors on breast cancer growth has, so far, resulted in conflicting evidence (both human and animal studies), IS has been shown to consistently elevate cancer risk and mortality (Hilakivi-Clarke et al., 1994; Sumis et al., 2016). In this regard, it has been recently suggested that socially isolated individuals may be characterized by a unique physiological *milieu* able to promote tumor growth (Hinzey et al., 2016). Thus, the aim of this study was to characterize the effects of four-month chronic social IS on tumor progression in female subjects of the MMTVNeuTg strain, a mouse model of brest cancer susceptibility. These mice overexpress the ErbB2 receptor, a condition observed in advanced breast cancers with an especially poor clinical prognosis (Slamon et al., 1987; Perou et al., 2000; Ursini-Siegel et al., 2007) thus, this model was selected because of its late latency of tumor occurrence, which allows studying the effects of chronic IS. We hypothesized that IS may activate the neuroendocrine and sympathetic nervous systems, leading to main changes in the hypothalamic expression levels of *Bdnf* and other stress-responsive mediators regulating mood and metabolism, such as Neuropeptide Y (*NpY*), Agouti-Related Peptide (*AgRP*), and Serum/Glucocorticoid-Regulated Protein Kinase 1 (Anacker et al., 2013; Baver et al., 2014). We focused on the hypothalamus, a brain area critical in the regulation of both energy balance and neuroendocrine activation. We also hypothesized that such stress-driven changes in the brain may, in turn, affect the production of mammary fat pad-derived adipokines and lead to depressive symptoms, eventually enhancing tumor progression. In this regard, mice were also scored for the occurrence of depressive-like behaviors by means of a saccharin preference and a forced swimming test (Berry et al., 2012). Neuroendocrine activation might greatly affect energy balance and promote fat browning (Razzoli and Bartolomucci, 2016; Razzoli et al., 2016). Indeed, metabolic reprogramming is a hallmark of cancer, and a growing body of evidence suggests that fat browning may be associated to a worst prognosis of different types of cancer (Hanahan and Weinberg, 2011; Huang et al., 2018). We also set to analyze the presence of fat browning as a marker of worst prognosis in the mammary fat pads of the MMTVNeuTg mice.

## Materials and Methods

### Animals

**Figure 1:**
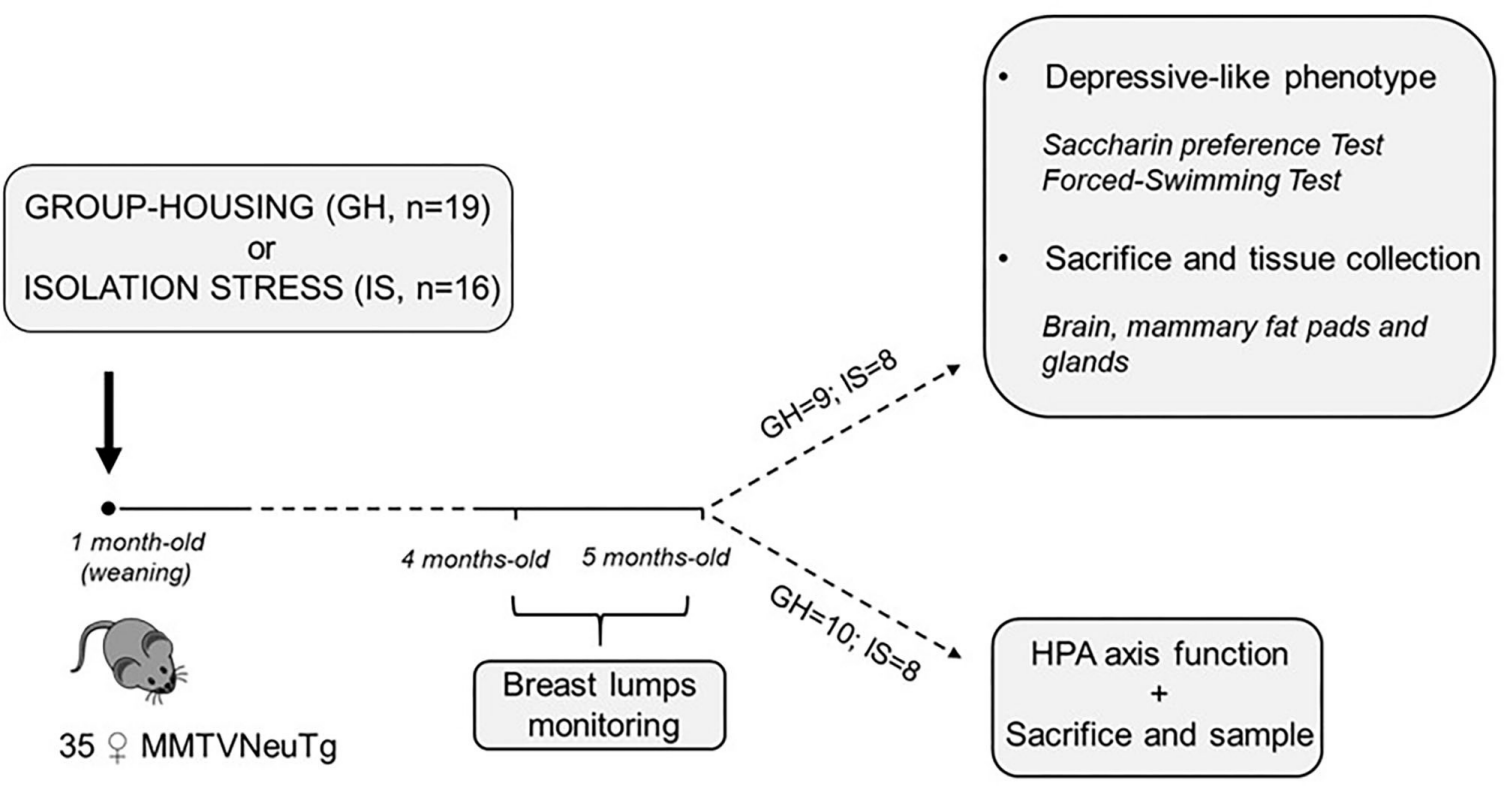
Timeline of the experimental design.

The experimental subjects were ErbB-2(Neu)TgMMTV-ErbB-2 (FVB background) mice purchased from The Jackson Laboratory (Bar Harbor, ME, United States) *via* Charles River (Calco, Italy). Upon arrival, all the animals were housed in the same room and provided with air conditioning (temperature 21 ± 1°C, relative humidity 60 ± 10%), in transparent Plexiglas cages (29 × 12 × 14 cm), under a reversed 12/12 h light/dark cycle with lights off from 0800 to 2000 h. Pellet food (standard diet Altromin-R, Rieper, Italy) and tap water were continuously available. More in detail, the MMTVNeuTg subjects developed hyperplasia at 5 months and focal adenocarcinoma and lung metastases at 7 months. The mice were used to fulfill the criteria of: (i) late latency of tumor occurrence to allow chronic stress treatment and (ii) extensively characterized tumor model with respect to morphological and histological features. The subjects in this experiment were 35 MMTVNeuTg female mice. At weaning, 16 of them were socially isolated (isolation stress, IS) to model long-term stress and, 19 were housed under standard laboratory conditions (group housing, GH−2 to 3 subjects/cage). Once a week, from the age of 16 weeks until week 20, each mouse was inspected for the presence of breast lumps. At this time point, approximately half of the subjects from each housing condition group were subjected to acute restraint stress (RS) to assess the functionality of the HPA axis (*n* = 10 MMTVNeuTg group-housed RS; *n* = 8 MMTVNeuTg isolated RS; see below for further details on the procedure). All the other subjects (*n* = 9 MMTVNeuTg group-housed control; *n* = 8 MMTVNeuTg isolated control) were assessed for depressive-like behavior by scoring the anhedonic profile (saccharin preference test) and learned helplessness (forced swimming test). At the end of the procedures, all the subjects were sacrificed to collect central and peripheral tissues in order to assess CORT levels (blood) and the gene expression of *Bdnf* , *SgK1, AgRP* and *NpY* (hypothalamus) as well as leptin and adiponectin and fat browning (in mammary gland fat pads) (see Figure 1).

All experimental procedures were reviewed by the ethical body of the Istituto Superiore di Sanità for animal welfare and conducted in conformity with the European Directive 2010/63/EU and the Italian legislation on animal experimentation, D.Lgs. 26/2014. They were authorized by the Italian Ministry of Health.

### Experimental Procedures

#### Restraint Stress

Following 4 months of isolation, half of the animals deriving from both the IS and GH (10 GH mice and 8 IS mice) underwent an acute RS procedure in order to assess the activity of the HPA axis. Each mouse was introduced in a conical 50-ml Falcon tube, provided with holes for breathing, adjusted on a laboratory bench with tape to prevent rolling. The stress was administered once and lasted three consecutive hours. To assess changes in CORT levels, blood samples were collected by tail nick at 0 (basal) 180 from the onset of stress, and after 240 min the mice were removed from the tube and sacrificed to collect trunk blood.

#### Saccharin Preference: Anhedonia

Mice have a strong preference for sweet solutions, such as those containing sucrose or saccharin, and the intake of these compounds is considered to result from their hedonic properties (Cryan and Holmes, 2005; Branchi et al., 2013). Twenty-week-old mice were habituated to a saccharin solution for 3 days. After this period, the experimental subjects underwent 10 days of familiarization, and each cage was provided with two bottles, one containing fresh tap-water and one containing 0.1% of saccharin solution. Bottles were daily weighed in order to monitor liquid consumption and switched to balance the effect of side preference in drinking behavior, which has been reported to be of importance for the correct evaluation of saccharin preference (Strekalova et al., 2004; Branchi et al., 2013).

Saccharin consumption was derived from bottle weight, and preference was evaluated on day 10 by looking at single mouse consumption for IS, while average cage-consumption was considered for GH. Saccharin preference was then calculated as follows:

$$\begin{array}{l} \text{\%saccharin preference}=\frac{\text{Saccharin solution intake}\times 100}{\text{water intake}+\text{Saccharin solution intake}} \end{array}$$

#### Forced Swimming Test: Learned Helplessness

The mice were tested according to the procedure developed by Porsolt et al. (1977). Each experimental subject was gently placed into a cylindrical glass (20 cm Ø, 40 cm height), filled with 25 cm of water at a temperature of 26 ± 1°C for 6 min for two consecutive days with dim light illumination (1 lux). When removed from the water, the mice were allowed to dry for 5 min under red light. Twenty-four hours later, a second session took place and latency, frequency, and duration of the following behavioral responses were scored: struggling (vigorous attempts at climbing the walls of the cylinder), swimming (active swimming around) and floating (total absence of movement).

#### Radioimmunoassay for Corticosterone Determination

Blood samples (20 μl, approximate volume) were collected individually in potassium EDTA coated tubes (1.6 mg EDTA/ml blood, Sarstedt, Germany). All samples were kept on ice and later centrifuged at 3,000 rpm for 15 min at +4°C. Blood plasma was transferred to Eppendorf tubes for CORT determination and stored at −20°C until further analysis. CORT was measured using a commercially available radioimmunoassay (RIA) kit containing 125 iodine labeled CORT; 5 μl of plasma was sufficient to carry out the CORT measurement. Sensitivity of the assay was 0.125 mg/dl, inter- and intra-assay variation was <10 and 5%, respectively (MP Biomedicals Inc., Santa Ana, CA, United States). Vials were counted for 2 min in a gamma-scintillation counter (Packard Minaxi Gamma counter, Series 5000).

#### Quantitative Real-Time Reverse Transcription–PCR

Total RNA was extracted from frozen hypothalamic and subcutaneous white adipose tissues with Trizol (Invitrogen, Carlsbad, CA, United States) and RNeasy Lipid Tissue Mini Kit (Qiagen, Hilden, Germany) following the instructions of the manufacturer. Quantity and quality were assessed with a Nanodrop ND-1000 spectrophotometer (Thermo Scientific, Wilmington, DE, USA) using OD260 for calculation of the concentration and the ratios 260/280 and 260/230 for assessing the purity of the samples. After cDNA synthesis using Superscript III (Invitrogen, Carlsbad, CA, United States), Real-time RT-PCR was performed with the TaqMan technology, using the ABI PRISM 7700 DNA Sequence Detection System (Applied Biosystems, Foster City, CA, United States). TaqMan reactions were carried out in 96-well plates using cDNA, TaqMan universal PCR mastermix, preoptimized, and preformulated TaqMan gene expressions assays that included specific primers and fluorescent probes for mouse, and water to a final volume of 25 μl according to the instructions of the manufacturer. Commercial ready-to-use primers/probe mixes (Assays on Demand Products, Applied Biosystems, Waltham, MA, United States) are listed: Adipoq #Mm00456425_m1; Lep #Mm00434759_m1; Insr #Mm01211875_m1; Bdnf # Mm 04230607_s1; Sgk1 #Mm 00441387_g1; Agrp #Mm 00475829_g1; Lepr #Mm 00440181_m1, Npy #Mm 03048253_m1; Eef2 #Mm 01171435_gh; GAPDH #Mm99999915_g1. Eef2 and GAPDH genes were used as internal controls for the efficiency of the RT-PCR assay and for the subsequent normalization of gene expression as assessed in the hypothalamus as well as in the adipose tissue, respectively. The Δct values were used for statistical analysis.

#### Assessment of Breast Lumps

The effects of prolonged social isolation (4 months) on the onset of breast lumps were assessed on female mice that were socially isolated from weaning until 5 months of age. Briefly, after a week from the arrival in the animal facility, the mice were either group housed (2–3 subjects/cage) or singly housed in cages with the following dimensions (29 × 12 × 14 cm). Once a week from the age of 16 weeks until week 20, each mouse was inspected for the presence of breast lumps through palpation of the 10 mammary glands. The inspection was carried out by trained personnel. The procedure for mammary gland monitoring and breast lumps detection in the MMTVNeuTg mice was adopted by other studies on ErbB2 transgenic mice with similar spontaneous carcinogenesis timing performed by the group of the authors or others (Boggio et al., 1998; Castiello et al., 2018). In particular, in order to limit operator-related biases, a very experienced technician took the responsibility of performing the procedure throughout the study duration.

#### Histology: Fat Browning and Proliferation Analysis

Hematoxylin and eosin staining (H&E) was performed on formalin fixed paraffin embedded (FFPE) tissue sections from the 5-month-old subjects (*n* = 5 for each experimental group). Slides (5 μm thick) were deparaffinized and hydrated through graded alcohols, and the H&E staining was performed according to standard protocols.

For immunolocalization studies, serial sections from two-mice-group of breast tissue were subjected to antigenic retrieval in Tris-EDTA pH 9 buffer, permeabilizated in 0.1% Triton X-100, saturated in 3% bovine serum albumin (BSA) at room temperature, and incubated with the rabbit anti- Ki-67 antibody (IHC-00375, Bethyl Laboratories, Inc. Montgomery, TX, United States) and subsequently with Alexa Fluor 555 conjugated anti-rabbit antibody (Molecular Probes, Eugene, OR, United States). Nuclei were stained with Hoechst 33342 (H3570, Invitrogen, Carlsbad, CA, United States). Negative controls were performed by omission of the primary antibody. Finally, the slides mounted with ProLong Gold Antifade (P36930, Invitrogen, Carlsbad, CA, United States) were analyzed by Olympus F1000 laser-scanning confocal microscopy (Olympus, Tokyo, Japan) (Maselli et al., 2019).

#### Statistical Analysis

Data were analyzed using parametric analysis of variance (ANOVA) with “social condition” (group-housed and isolation stress) as between-subjects factor and “time course” as within-subject repeated measures (CORT assessment; breast lumps). For outcomes that did not follow a normal distribution (*Bdnf* , *NpY, SgK1* and *AgRP*) data were normalized by transforming raw data into their square root. *Post-hoc* comparisons were performed using the Tukey's test. Statistical analysis was performed using Statview II (Abacus Concepts, Berkeley, CA, United States). Data are presented graphically as means ± SEM and as box plot (observations outside the ranges are represented with dots outside the boxes). A significance level of 0.05 was chosen.

The raw data supporting the conclusions of this article will be made available by the authors, without undue reservation.

## Results

### HPA Axis Assessment

**Figure 2:**
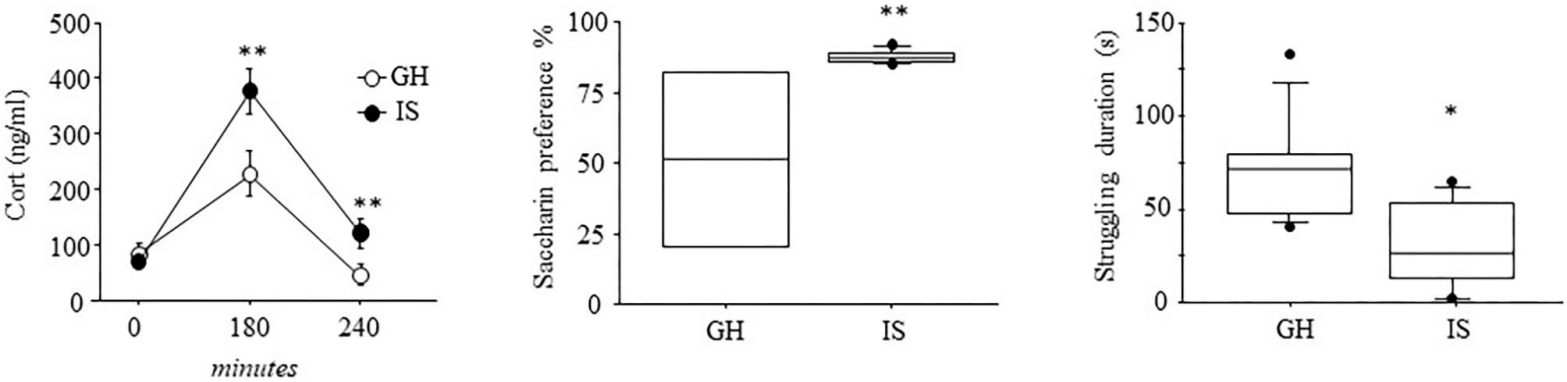
Neuroendocrine and depressive-like behavioral profile of the MMTVNeuTg mice. Three hours of acute restraint stress resulted in higher CORT levels in the MMTVNeuTg female mice that underwent 4 months of chronic isolation stress, suggesting a stronger activation of the neuroendocrine system. The isolated mice were also characterized by elevated saccharin consumption in a saccharin preference test, as well as by reduced struggling duration, suggesting a depressive-like behavior. Data are presented graphically as means ± SEM box plots (observations outside the ranges are represented with dots outside the boxes). *p < 0.05; **p < 0.01. Number of subjects for CORT: 8 (IS); 10 (GH); for saccharin preference: IS (7) and GH (4); for forced swim stress IS (8); GH (9).

When the mice were challenged with an acute RS to assess neuroendocrine reactivity following a chronic IS procedure, a main effect of the social conditions was observed, showing overall increased CORT levels in the IS group [*F*_(1, 16)_ = 11.223; *p* = 0.0041]. More in detail, while basal levels did not differ between IS and GH mice, a significant interaction between social condition and time course revealed that IS resulted in a specific increase in CORT levels both at 180 and 240 min from the beginning of the RS procedures *F*_(2, 32)_ = 4.394; *P* = 0.0206 (*post-hoc* comparison *P* < 0.05, see Figure 2).

### Saccharin Preference: Anhedonia

When the hedonic profile of female mice that experienced 4 months of social isolation was characterized, we observed an elevated preference toward saccharin consumption in the IS group when compared with the GH condition [*F*_(1, 9)_ = 7.844; *p* = 0.0207], confirming that prolonged social isolation influences response to stimuli associated with reward as previously shown (Berry et al., 2012).

### Forced Swimming Test—Learned Helplessness

Assessment of the learned helplessness profile, as a result of IS, showed that the female mice, isolated for 4 months, were characterized by a specific decrease in the frequency and duration of struggling, an escape-like behavior indicative of a proactive coping strategy toward stress [*F*_(1, 15)_ = 9.022; *p* = 0.0089; *F*_(1, 15)_ =10.204; *p* = 0.0060, respectively for frequency and duration]; latency to display this behavior did not differ between IS and GH [*F*_(1, 15)_ = 1.072; *p* = 0.3169]. Swimming behavior was also affected by IS, since these mice showed a decreased number of swimming bouts [frequency: *F*_(1, 15)_ = 5.093; *p* = 0.0394)]. However, latency and duration were not affected by chronic stress [*F*_(1, 15)_ = 0.821; *p* = 0.3792; *F*_(1, 15)_ = 3.371; *p* = 0.0863]. The IS and GH mice did not differ as for floating behavior [*F*_(1, 15)_ = 0.132; 0.247; 1.032; *p* = 0.7212; 0.6267; 0.3258, respectively, for latency, frequency, and duration; see Figure 2].

### Hypothalamic Gene Expression in Response to Stress

**Figure 3:**
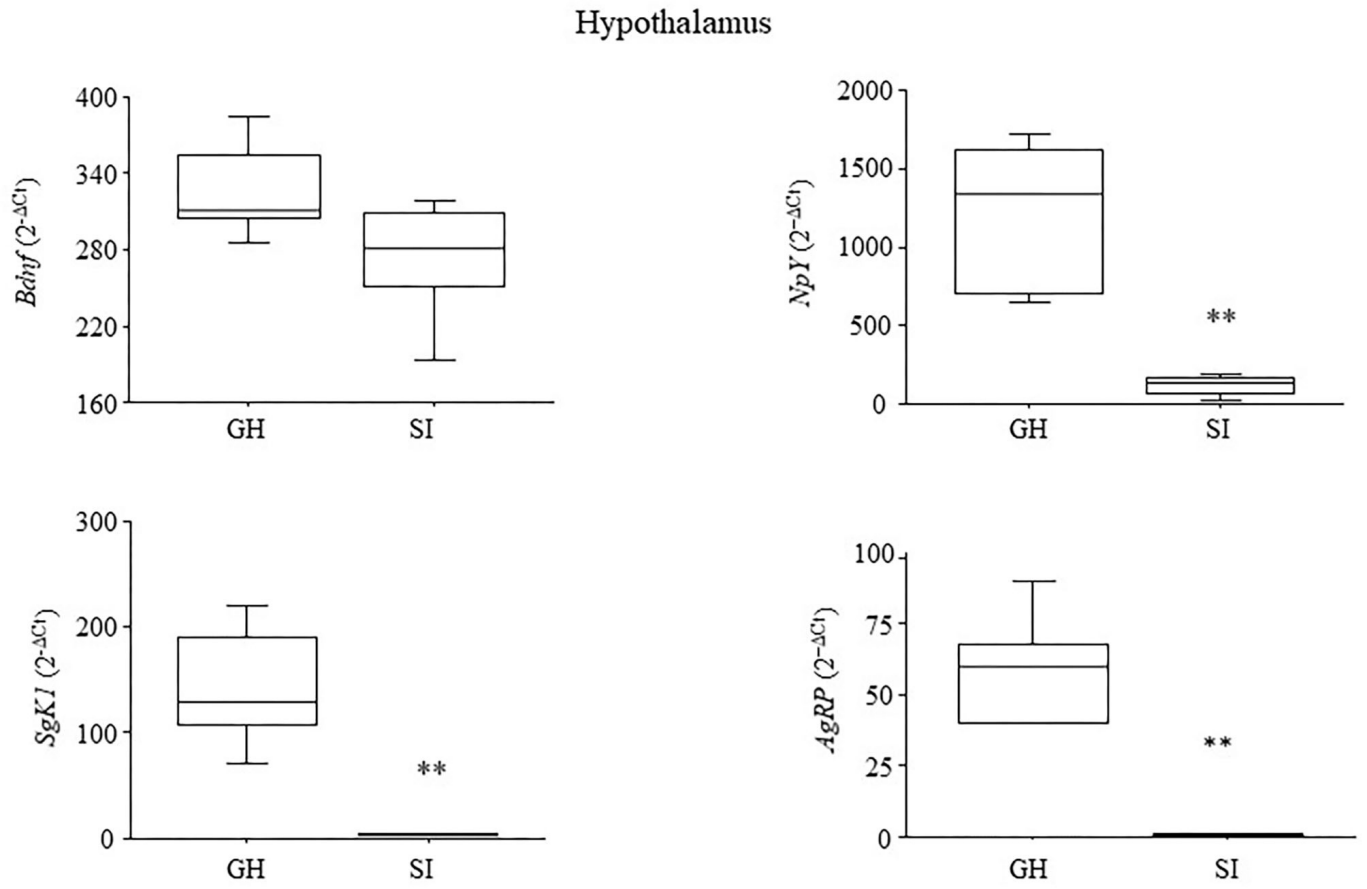
Hypothalamic gene expression. Chronic social isolation stress resulted in the decrease in the expression levels of genes specifically involved in stress reactivity (*SgK1*), and metabolism (*NpY* and *AgRP*). Expression of the neurotrophin *Bdnf* was somewhat reduced but not significantly after 4 months of IS. Raw data were transformed using the square root and are presented graphically as means ± SEM box plots (observations outside the ranges are represented with dots outside the boxes). **p < 0.01. Number of subjects: 4–5 within each experimental group.

At 5 months of age, a time when tumor progression manifests in the MMTVNeuTg mice, *Bdnf* levels did not differ between the IS and GH subjects [*F*_(1, 8)_ = 3.698; *P* = 0.907]. In contrast, the IS mice were characterized by decreased levels of *NpY* [*F*_(1, 8)_ = 23.784; *P* = 0.0012], *SgK1* [*F*_(1, 7)_ = 22.893; *P* = 0.002] and *AgRP* [*F*_(1, 8)_ = 39.056; *P* = 0.0002]; see Figure 3.

### Gene Expression in Adipose Tissue

**Figure 4:**
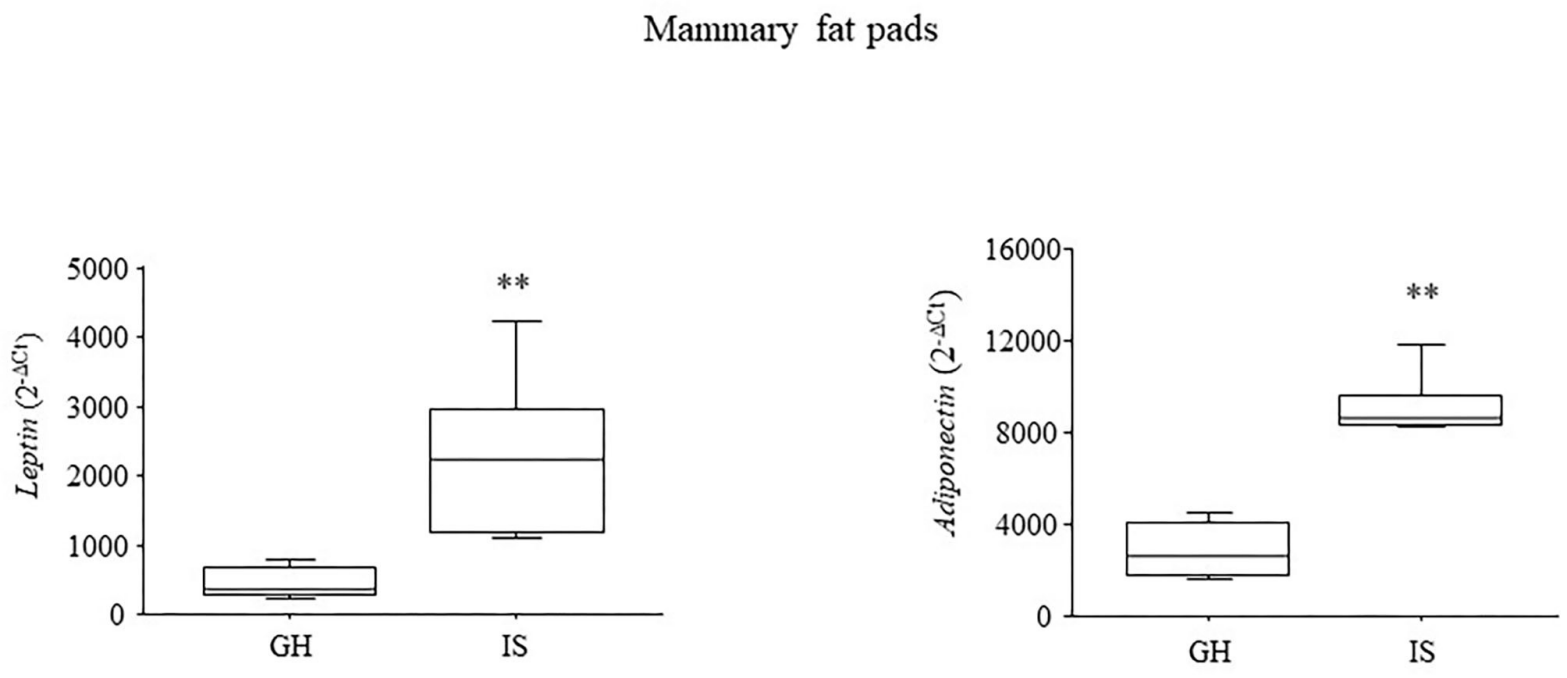
Adipokines gene expression in the mammary fat pads. Chronic social isolation stress increased the levels of leptin and adiponectin in the MMTVNeuTg female mice. Data are presented graphically as means ± SEM box plots (observations outside the ranges are represented with dots outside the boxes). **p < 0.01. Number of subjects: 4–5 within each experimental group.

At 5 months of age, IS increased both the *leptin* and *adiponectin* gene expressions in the mammary fat pads [*F*_(1, 8)_ = 9.768; *P* = 0.0141; *F*_(1, 8)_ = 52.326; *P* < 0.0001, respectively, for leptin and adiponectin]; see Figure 4.

### Assessment of Breast Lumps

**Figure 5:**
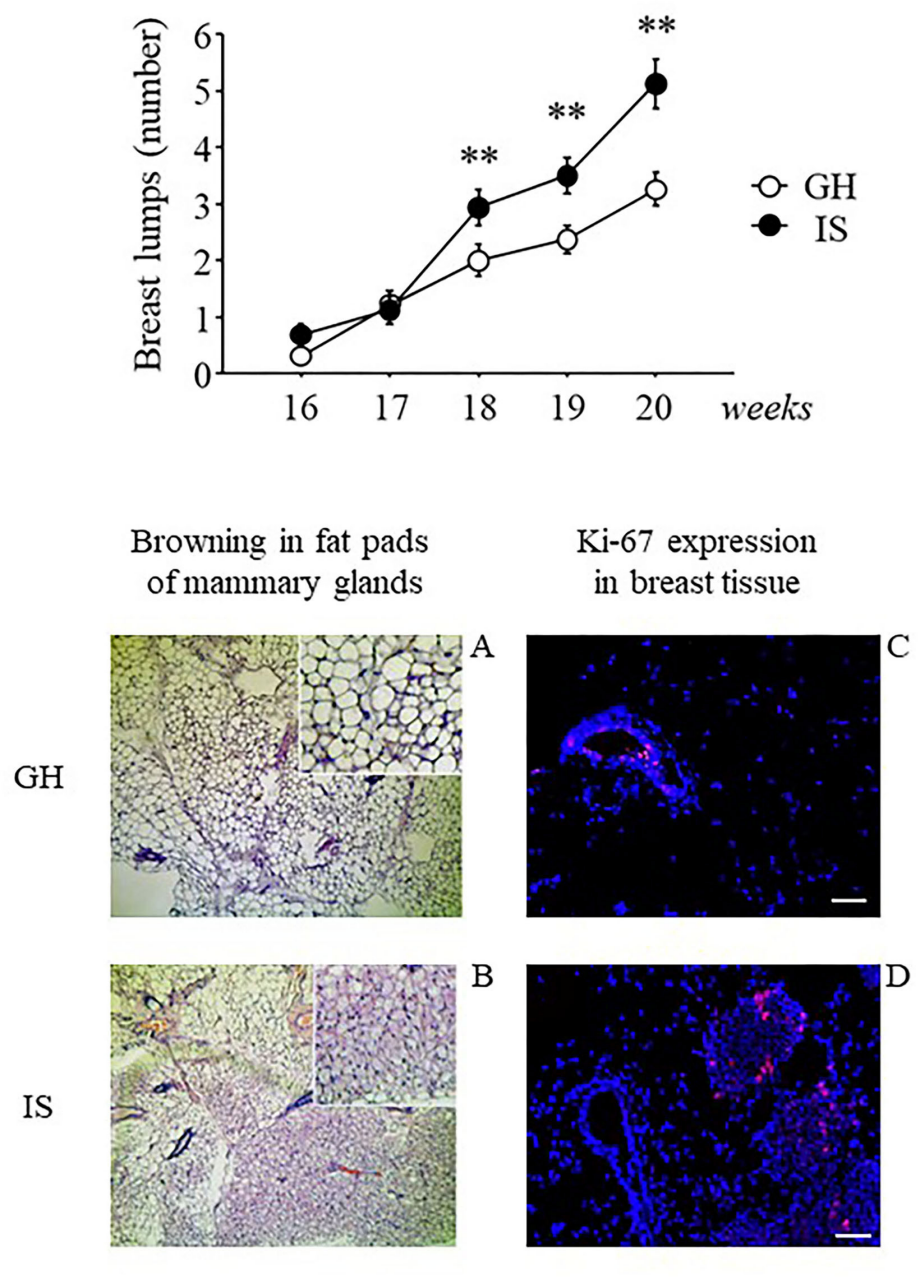
Breast lumps and histological analysis of breast tissues from the MMTVNeuTg female mice. Breast lumps and fat browning in the fat pads of the MMTVNeuTg female mice. Isolation stress resulted in a time-dependent increase in the number of breast lumps in the breast cancer susceptibility MMTVNeuTg mouse model, suggesting an acceleration in tumor onset (upper panel). Moreover, when compared with the GH condition (lower panel, **A** and **C**) chronic IS promoted the development of brown adipose tissue and was associated with a greater number of Ki-67+ cells (red nuclei) in the mammary gland (lower panel, **B** and **D**). Data are presented graphically as means ± SEM (upper panel). *Post-hoc* comparisons: **p < 0.01. Number of subjects: IS (16); GH (19). Lower panel: representative sections from mammary fat pads (**A** and **B**) and mammary gland (**C** and **D**), immunostained with Ki-67 (red) and counterstained with Hoechst (blue). Scale bar = 50 μm.

Twenty weeks of social isolation resulted in a greater number of breast lumps compared with group-housed controls [main effect of the social condition: *F*_(1, 33)_ = 8.325; *p* = 0.0068]. This effect was particularly apparent starting from weeks 18 to 20 [interaction between housing condition and time course: *F*_(4, 132)_ = 6.696; *p* < 0.0001; *post-hoc* comparisons *p* < 0.01 IS v. GH at weeks 18, 19, and 20; see Figure 5]. To exclude the effects of hormonal fluctuations on breast lump appearance, the subjects were inspected to assess estral cycle phase just before the beginning of RS, and no difference was observed in vaginal smear between the groups (data not shown).

### Qualitative Analyses on Breast Tissues

Histological examination of the mammary glands indicated that prolonged exposure to chronic IS induced WAT “browning” in the mammary fat pads with adipocytes being characterized by multilocular small lipid drops. In contrast, the WAT tissue of the GH mice was characterized by cells with a single (unilocular) large lipid drop (Figure 5, lower panel, A and B). Moreover, when compared with GH, analysis by confocal microscopy revealed that the IS mice were characterized by greater Ki-67+ cells (red nuclei), suggesting greater cellular proliferation (Figure 5, lower panel, C and D).

## Discussion

The main aim of this study was to characterize the effects of chronic social isolation stress (IS)—started soon after weaning—on the crosstalk between brain and mammary fat pads in a mouse model of breast cancer. The results show that IS increases neuroendocrine responsiveness to further stressors and lead to important changes in the hedonic profile (saccharin preference test) and copying strategies (forced swimming test); this behavioral phenotype is accompanied by a strong downregulation of hypothalamic genes involved in stress and metabolic regulations, such as *NpY, AgRP* and *SgK1*. Moreover, and most importantly, the mammary fat pads of the IS mice show elevated adipokines expression levels and an increased amount of BAT. These changes were associated to an earlier appearance of breast lumps in our MMTVNeuTg breast cancer model.

Chronic social isolation did not affect basal CORT levels but resulted in increased HPA axis activation following an acute restraint stress challenge. These changes in the neuroendocrine response were accompanied by a depression-like phenotype. Indeed, the IS mice showed increased saccharin consumption, in addition to a decrease in the frequency and duration of struggling and in the duration of swimming behavior, as assessed in the forced swimming test. These results are in agreement with the previous data showing that 3 weeks of IS, in male mice, lead to HPA axis hyper-responsiveness to acute challenges, and this was associated to an increase in the consumption of a sweetened solution (Berry et al., 2012). Anhedonia is a core symptom of depression relying upon a reduced ability to feel pleasure from everyday life experiences (Rizvi et al., 2016). A very common method to measure anhedonia in laboratory rodents is by assessing the preference for the consumption of sweetened solutions. However, this measure is tricky, and the significance of the stress-induced preference for saccharin is debated, being highly dependent on the type of stress (physical vs. emotional) as well as by the metabolic set point of the animals (Adam and Epel, 2007). Worth to notice, Ulrich-Lay et al. provided evidence that saccharin consumption may reduce neuroendocrine activation, suggesting that the rewarding properties of saccharin sweet taste lead to a stress-buffering effect (Ulrich-Lai et al., 2010). Thus, the increase in saccharin preference that we observed in this and the previous study might represent a coping strategy to dampen the stress resulting from the chronic IS condition (Berry et al., 2012). In the forced swimming test, the IS mice showed a decrease in struggling and swimming behaviors, a condition clearly mirroring the shift from an active to a more passive coping strategy, overall suggesting the expression of a depressive-like behavior. Although we did not observe differences in floating between the IS and the GH mice, we cannot exclude that by re-exposing the animals to a second session (like in the standard paradigm of this test) we could have also observed an increase in this behavior.

Stress begins with the perception and interpretation of everyday challenges, leading to plastic changes in the brain and peripheral functions that may affect disease onset/progression (McEwen, 2012; Borgi et al., 2020). The hypothalamus is the brain region capable of integrating different inputs from the external environment, thus the analysis was focused on hypothalamic genes previously shown by Cao et al. to be activated in animal models of cancer following environmental enrichment. Interestingly, and as hypothesized, we found decreased levels of those same genes found to be activated upon social/environmental enrichment by these authors (Cao et al., 2010). More in detail, while we found a specific decrease in *SgK1, AgRP*, and *NpY*, no change was observed upon *Bdnf* assessment. This neurotrophin is highly expressed in the hypothalamus, and its transcription is complex and shows great variation across different nuclei (Timmusk et al., 1993; Chen et al., 2003; Miranda et al., 2019). However, we measured BDNF expression levels in the whole hypothalamus, and this might have reduced the chance to appreciate significant differences.

Levels of *SgK1* were decreased in the hypothalamus of our mouse model of breast cancer when subjected to IS. This gene is involved in many important GC-dependent functions affecting both stress and metabolism (Ullrich et al., 2005; Van Gemert et al., 2006; Anacker et al., 2013; Deng et al., 2018). Decreased *SgK1* in the prefrontal cortex has been associated to stress-related behavioral and morphological phenotypes in both human and animal studies (Licznerski et al., 2015). Moreover, decreased hypothalamic levels of *SgK1* were observed upon dexamethasone infusion (a selective GR agonist) and were associated to increased adiposity without changes in body weight, suggesting that this molecule may represent a link between emotional distress and fat metabolism (Deng et al., 2018).

*AgRP* and *NpY* are powerful orexigenic neuropeptides that play a main role in energy homeostasis; within the central nervous system they are expressed primarily in the hypothalamus and are (down-) regulated by peripheral nutritional factors that include fat-secreted leptin (Mizuno et al., 2003). Recently a role for *AgRP* has been proposed in the orchestration of the complex interaction among stress, food reward and ingestive behavior (Fang et al., 2021) such that when *AgRP* neuronal activity is impaired, neural circuits sensitive to emotion and stress (pro-opiomelanocortin—POMC) are engaged and finely modulate food palatability (Denis et al., 2015; Morales and Berridge, 2020). Indeed, our data show that the IS mice were characterized by reduced hypothalamic *AgRP* expression levels and increased consumption of saccharin. This piece of data suggests that chronic IS may change individual incentive-sensitization in favor of palatable food rewards (Denis et al., 2015; Morales and Berridge, 2020; Fang et al., 2021). Another important player in these regulations is *NpY*, which, beyond its main role in the control of metabolism and food intake, is also involved in stress coping strategies and in the pathophysiology of depression. Indeed, clinical studies show that increased plasma NPY levels correlate with improved coping abilities (Morgan et al., 2000; Kautz et al., 2017). In contrast, in pathological conditions, such as depression, NPY release is reduced (Redrobe et al., 2002). Animal studies confirm and strengthen the above-mentioned evidence showing, for example, that exposure to chronic variable stress reduces NPY levels within the amygdala (McGuire et al., 2011), while animals being characterized by more adaptive coping strategies show higher *NpY* levels in different brain areas (Hawley et al., 2010). Moreover, intracerebroventricular administration of *NpY* in rats significantly reduces immobility time in the forced swimming test in a dose dependent fashion (Redrobe et al., 2002). This piece of data is particularly interesting, since we observed a reduction in struggling and swimming in the forced swimming test in association with reduced hypothalamic *NpY*, suggesting that this neuropeptide might, at least in part, account for the passive coping strategy observed in this test.

Chronic activation of the stress machinery leads to an allostatic load powerfully affecting both the central nervous system and peripheral organs and tissues; thus, pathological changes in mood and emotionality are often associated to changes in fat metabolism and body weight. We found that the fat pads of the IS mice were characterized by increased leptin and adiponectin expression levels, an effect associated to increased BAT and to an overall earlier appearance of breast lumps, in the absence of significant changes in body weight (data not shown). The data are in line with the findings of Sun et al. who found increased circulating leptin levels and overall increased adiposity (with regard to both WAT and BAT) in IS mice (Sun et al., 2014). Likewise, Volden et al. (2013) found a specific upregulation of leptin levels in the mammary fat pads of a mouse model of breast cancer subjected to IS. The mammary gland is a dynamic organ, mainly composed of fat tissue, that changes its architecture and function in response to hormonal and neuroendocrine triggers. Elevated leptin levels have been observed in macrophage-infiltrated WAT characterized by inflammation and hypertrophy of the adipocytes (Armani et al., 2017). This pro-inflammatory condition is usually associated to decreased adiponectin levels, an anti-inflammatory adipokine, whose blood levels are inversely related to breast cancer risk in patients (Yu et al., 2019). Here, we found increased levels of adiponectin in the WAT of the IS mice, possibly suggesting a compensatory mechanism in the attempt to restore metabolic homeostasis in the cancer-prone model.

The same studies previously mentioned (Sun et al., 2014) also found important changes in BAT following isolation stress, a result that we also report. This fat tissue is characterized by small numerous adipocytes and a large number of well-developed mitochondria that facilitate the catabolism of lipids for heat production (Carpentier et al., 2018). Wang et al. found that the browning of mammary fat, in the proximity of malignant breast tumors, was greater than that close to benign lesions in a cohort of Chinese women (Wang et al., 2014). Thus, although the mice were sacrificed before the appearance of tumor masses, and we are well aware that breast lumps may not be directly associated to malignant lesions (Daly and Puckett, 2020), we can hypothesize that BAT development in the IS mice might stand for a more aggressive pro-tumorigenic micro-environment. In this context, it is worth to notice that BAT appearance, as a result of isolation stress, holds many implications for cancer pathology, since the phenomenon of fat browning has been associated to negative energy balance as observed during cachexia (a syndrome characterized by uncontrolled weight loss, muscle atrophy, fatigue, and weakness) (Caron et al., 2018). There is also evidence to suggests that leptin feedback on POMC neurons may directly promote fat browning through the activation of the sympathetic nervous system (Dodd et al., 2015; Caron et al., 2018). At the same time, the inhibition of *NpY/AgRP* signaling by leptin may drive emotional responses and activate the reward system (in the attempt to buffer stress) through the activation of POMC neurons (Baver et al., 2014; Denis et al., 2015; Fang et al., 2021).

## Conclusions and Future Perspectives

**Figure 6:**
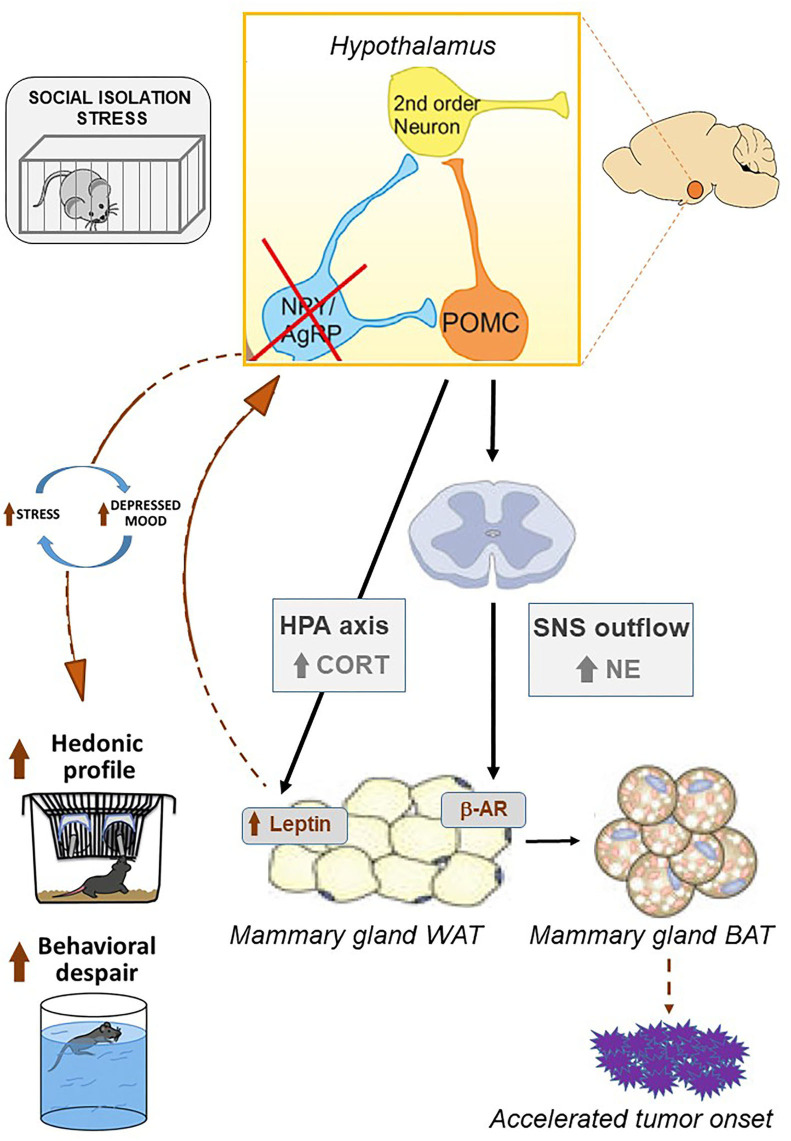
Chronic social isolation stress results in the activation of the hypothalamic-pituitary-adrenal axis. Upon the activation of the HPA axis, corticosterone acts directly on the white adipose tissue by increasing leptin levels. This pro-inflammatory adipokine reaches target neurons in the hypothalamus, resulting in the inhibition of *NpY/AgRP* signaling that in turn increases the neuronal activity of POMC (Baver et al., 2014; Denis et al., 2015; Fang et al., 2021). As a result, an increase in reward-driven saccharin consumption is observed as well as passive coping strategies in the forced swimming test. The appearance of depressive-like behaviors promotes a feed-forward loop promoting chronic stress. Likewise, hypothalamic signaling *via* POMC neurons triggers the activation of the sympathetic nervous system that, by secreting norepinephrine, reaches beta-adrenergic receptors within WAT, promoting the development of brown adipose tissue (Dodd et al., 2015; Caron et al., 2018). BAT is characterized by increased metabolic rate and may, in turn, accelerate tumor onset in the MMTVNeuTg mouse model. Picture modified from Caron et al. (2018), Denis et al. (2015), and Franklin et al. (2012).

This study has some limitation that is mainly linked to the specific animal model, which is a mouse transgene that only partially reproduces the complexity of cancer onset and progression. Furthermore, we were limited in the number of subjects used. Nonetheless, we suggest that these data are important as they point to IS as a pervasive stressor that is able to specifically target and affect neuronal circuits, mastered by the hypothalamus, modulating mood, stress-reactivity, and energy homeostasis. The activation of such IS-driven machinery may hold main implications for the onset and maintenance of pro-tumorigenic environments (see Figure 6).

Recent evidence has suggested that the efficacy of existing cancer therapies is impaired by chronic stress, because it prevents the immune system from responding properly (Zhang et al., 2020). Moreover, the metabolic requirements of immune cells in the tumor microenvironment greatly influence the success of immunotherapy such that the use of metabolic modulators has been proposed as a potential strategy to “support energetic rewiring of immune cells that might boost their anti-tumor capacity” (Guerra et al., 2020). Thus, from a translational perspective, beyond primary strategies to counteract social isolation and to promote social support, tailored dietary strategies and lifestyle interventions that should include physical activity (Cui et al., 2019) hold the potential to improve not only individual metabolic set point but also mood, ultimately targeting the stress-immune-cancer axis.

Sex and gender represent primary variables affecting different elements of the stress process, such as how a stressful event is perceived, as well as coping responses. A growing body of research has shown striking sex biases in stress-related anxiety and mood disorders with women more likely than men to develop depression during their lifetime. Thus, a special attention should be given to the appraisal of stressors of women (Borgi et al., 2020).

## Data Availability Statement

The raw data supporting the conclusions of this article will be made available by the authors upon request without undue reservation.

## Ethics Statement

The animal study was reviewed and approved by Ethical Body of the Istituto Superiore di Sanità for Animal Welfare and conducted in conformity with the European Directive 2010/63/EU and the Italian legislation on animal experimentation, D. Lgs. 26/2014. They were authorized by the Italian Ministry of Health.

## Author Contributions

AB and BC analyzed, interpreted data, and wrote the manuscript. SCa collected all behavioral data. MTD and EA provided their expertise in the mouse model of breast cancer. SCe and PS carried out immunohistochemistry of BAT. CR and EO carried out gene expression. RP and GP carried-out the ki-67 analysis on breast tissue, interpreted data, and drafted the revised version of the manuscript. FC designed the experiment and provided data interpretation. All authors contributed to the article and approved the submitted version.

## Conflict of Interest

The authors declare that the research was conducted in the absence of any commercial or financial relationships that could be construed as a potential conflict of interest.
